# Decreased Functional Connectivity Between the Right Precuneus and Middle Frontal Gyrus Is Related to Attentional Decline Following Acute Sleep Deprivation

**DOI:** 10.3389/fnins.2020.530257

**Published:** 2020-12-21

**Authors:** Bozhi Li, Liwei Zhang, Ying Zhang, Yang Chen, Jiaxi Peng, Yongcong Shao, Xi Zhang

**Affiliations:** ^1^Department of Neurology, The Second Medical Center, National Clinical Research Centre for Geriatric Diseases, Chinese PLA General Hospital, Beijing, China; ^2^Key Laboratory of Behavioral Science, Institute of Psychology, Chinese Academy of Sciences, Beijing, China; ^3^Department of Psychology Medical, The Eighth Medical Center, Chinese PLA General Hospital, Beijing, China; ^4^School of Biological Science and Medical Engineering, Beihang University, Beijing, China; ^5^Beijing Advanced Innovation Centre for Biomedical Engineering, Beihang University, Beijing, China; ^6^School of Psychology, Beijing Sport University, Beijing, China

**Keywords:** attention, functional connectivity, middle frontal gyrus, precuneus, sleep deprivation

## Abstract

**Objectives:**

Acute sleep deprivation (SD) seriously affects cognitive functions, such as attention, memory, and response inhibition. Previous neuroimaging studies have demonstrated a close relationship between the functional activities of the precuneus (PC) and the function of alert attention. However, the specific effect of the PC on attention decline after acute SD has not been elucidated. In this study, we used resting-state functional magnetic resonance imaging (fMRI) to study the relationship between the changes of the PC functional connectivity and alertness decline after total SD.

**Methods:**

Thirty healthy, right-handed adult men participated in the experiment. Alert attention and functional connectivity were assessed by the Psychomotor Vigilance Test and a resting-state fMRI scan before and after total SD. The region of interest to region of interest (“ROI-to-ROI”) correlation was employed to analyze the relationship between the PC and other brain regions after acute SD.

**Results:**

Participants showed decreased alert attention after total SD. In addition, SD induced decreased functional connectivity between the right PC and the right middle frontal gyrus (MFG). Moreover, there was a significant correlation between the decreased PC functional connectivity and alertness decline after total SD.

**Conclusion:**

Our findings suggest that the interruption of the connection between the right PC and the right MFG is related to the observed decline in alert attention after acute SD. These results provide evidence further elucidating the cognitive impairment model of SD.

## Introduction

Modern society and occupational demands have led to increasing sleep deprivation (SD). Due to long and irregular working and studying hours, both adults and adolescents have been getting less sleep over the past three decades (Ford et al., 2015; Keyes et al., 2015; Sheehan et al., 2018; Hisler et al., 2019). Moreover, about 35% of the population sleep less than 6 h/day due to tight work schedules and the use of electronic devices before bedtime. Short duration of sleep is significantly associated with increased mortality (Itani et al., 2017), and SD has significant effects on body function. Some believe that SD influences higher brain functions, such as mood and working memory (Krause et al., 2017; Krause Posada-Quintero et al., 2019), and basic brain functions, such as attention and alertness. SD is not simply a representation of SD and its associated functions; it is also a combination of many harmful factors, such as prolonged insomnia and lack of sleep (Krause et al., 2017).

Extensive research has been carried out on the effects of SD on attentional alertness. Molecules associated with sleep stress, such as adenosine and the hypothalamic system, control the mechanisms involved in the transition between sleep and arousal and are candidates for the chemical signaling and network regulation that typically regulates sleep loss in dose-dependent attention disorders (Saper et al., 2010). Neuroimaging analysis of how acute SD alters brain function related to attention tasks has shown that functional magnetic resonance imaging (fMRI) signals in the dorsolateral prefrontal cortex (DLPFC) and the parietal sulcus were reduced during attention tasks after SD (Chee et al., 2010, 2011; Czisch et al., 2012). In fact, not only did SD reduce task-related activity in these frontal and parietal regions, but it also reduced connections to the lateral visual cortex during visual–spatial attention tasks. In addition, SD affects thalamic activity during sustained attention, suggesting that the thalamus may play an interactive role in the SD-influenced network (Tomasi et al., 2009; Chee et al., 2011). Recently, default mode network (DMN) instability was discovered in the attention deficit associated with SD (Buckner and DiNicola, 2019). Some reports describe the inability of the anterior and posterior cortical regions of the DMN to completely deviate from the midline during the execution of sustained attention tasks under SD conditions. In addition, in the sustained attention test, increased DMN activity during task execution predicted slower execution speed and decreased accuracy of participants (Drummond et al., 2005). In contrast, the significance detection network, including the frontal insular cortex, showed reduced activity during attentional tasks after sleep loss (Ma et al., 2015).

Areas of the brain that belong to the DMN are more susceptible to SD (Chen et al., 2018; Tashjian et al., 2018). It is reported that the precuneus (PC) may be an important “distribution node” in the DMN (Li et al., 2019). Furthermore, based on partial correlation analysis, Fransson pointed out that the PC might be the only network node in the DMN that directly interacts with other nodes (Fransson and Marrelec, 2008). A growing body of other evidence suggests that in clinical and laboratory conditions, the PC is closely related to cognitive functions, such as attentional alertness and neuropsychiatric activities (Cherkassky et al., 2006; Groen et al., 2009). Studies have shown that the PC and the neighboring posterior cingulate cortex are responsible for ongoing information gathering from ourselves and the world around us and automatically distributing it (Cabeza and Nyberg, 2000). In the resting state, the PC and the cingulate cortex, as parts of the DMN, are active and are involved in a wide range of attention processes (Hutchinson et al., 2009; Vogt and Derbyshire, 2009). In addition, the structural and functional connections between the PC and thalamus are consistent with the white matter pathways between the PC and thalamus (Crone et al., 2015; Hannawi et al., 2015; Cunningham et al., 2016). Indeed, the role of the PC is not well established due to the lack of specific studies on its function. As it is located between the somatosensory and visual cortex and has no special functional role, the PC has not been the subject of profound research (Cavanna and Trimble, 2006). Until now, the structural and functional changes of the PC under SD have rarely been studied, and the analysis of functional connectivity of the PC by fMRI and attentional alertness has not been analyzed.

In light of this, we attempted to explore the changes in the functional connections between the PC and other brain regions during SD, as well as the correlation between the changes of functional connectivity and in certain aspects of cognitive functioning. In order to screen and preliminarily verify functional changes in connections between the PC and other brain regions, we designed a variety of Visual Analog Scale (VAS) to initially identify the possible brain functions (Huang et al., 2019) and performed further studies using the Psychomotor Vigilance Test (PVT), which is regarded as a “gold standard” tool to assess for the neurobehavioral consequences of SD (Basner and Dinges, 2011; Basner et al., 2015). Through these methods, we further define the functions of the PC.

## Materials and Methods

### Participants

We recruited a total of 30 young adults (30 male, right-handed, age range: 20–30 years) and provided financial compensation for their participation in this study. The subjects were all undergraduates or graduate students. Informed consent forms were signed voluntarily by the participants after the process, risks, and benefits of the study were explained to them in detail. After enrollment, specialist physicians who were qualified to practice medicine in China performed standardized medical examinations on them. The main forms of medical examination included subjective inquiry confirmed by self-report and objective examination (i.e., electrocardiogram, scales, hematological monitoring) to eliminate potential major diseases. The inclusion criteria were: (1) No history of cardiovascular disease, respiratory system, nervous system, infectious diseases, mental disorders, and sleep disorders and (2) Having regular daily life and rest habits without sleep disorders (Pittsburgh Sleep Quality Index scale <7); 1 week before the study began, daily activities of participants were conducted according to the normal routine, and consumption of stimulant drinks and food, such as carbonated drinks, tea, and coffee, was banned, and smoking was recommended to be avoided. All subjects participated in this study voluntarily and provided written informed consent before participation. This study was approved by the Research Ethics Committee of Beihang University (Beijing, China).

### Behavioral Measures

As an auxiliary means of detection, the VAS, a validated and simple psychometric tool, was used to assess the levels of alertness, anxiety, attention, self-confidence, anger, and nervousness of the participants prior to and after SD (Huang et al., 2019). In order to adapt to the 100-point system of the Chinese people, we adjusted the VAS slightly to an evaluation scale that increased every 10 points and is divided into 10 grades from 0 to 100, corresponding to the 0–10 scale of the standard VAS. Through this, we tried to evaluate this simple measurement method and perform a preliminary assessment.

The PVT–a classic monitoring tool for levels of psychomotor vigilance–was used to measure certain aspects of cognitive functioning reflecting the neurobehavioral consequences of SD (Basner et al., 2011). Subjects were instructed to press the button as soon as they saw a visual stimulus presented at random inter-trial intervals appearing on the screen while trying to minimize error operation. A dot (diameter 3 1/4 cm, viewing angle 1.5 × 1.5°) appeared as a visual stimulus in the center of an LCD screen with 1,024 × 768-pixel resolution (refresh rate, 60 Hz). The red dots appeared pseudo-randomly in the center of the screen, lasting up to 1,000 ms before subjects pressed the button, and disappeared immediately when the button was pressed.

### Procedures

The experiments were conducted in a sleep laboratory at the General Hospital of People’s Liberation Army and Institute of Beihang University (Beijing, China), which included a sleep monitoring room with noise less than 30 dB and a daily activity room. During the process of the experiment, the subjects took part in the study in batches of four people each, and two operators monitored the physical and mental states of the subjects and supervised the relevant experimental contents. At 8:00 a.m. on the first day of the experiment, the subjects arrived at the laboratory and wore body-movement watches. They performed daily activities from 8:00 to 20:00, including playing games, reading, talking, sitting for rest, and eating. The collection of experimental data was also accomplished during this time. From 22:00 to 8:00 on the second day, the subjects completed at least 8 h of sleep under the supervision of the operators, with monitoring of body movement during sleep. SD began at 8:00 on day 2 and ended at 20:00 on day 3. During the study, subjects performed normal daily activities and completed relevant experimental data collection. MRI scans began at 20:00 on the third day. During this time, monitoring activities, such as electrocardiography and the subjective assessment of scales, were completed. At the end of the experiment, the subjects were supervised by the experimenters while having a restorative-free sleep in the sleep laboratory. After completing the physical health status assessment on the morning of the fourth day, the subjects left the sleep laboratory at 12:00. During the whole experiment, it was ensured that no less than one operator was medically qualified.

Comparisons before and after the experiment design were carried out with the official start of the test. All subjects underwent scanning twice: one during 36 h of SD and another during rested wakefulness (RW). The two scans were performed at least 3 weeks apart to minimize the possibility of residual SD side effects in participants who had undergone SD scans prior to RW scans. Both scans were performed at the same time of day using the same scanning sequence.

All participants were scanned in a 3.0 T Siemens Magnetom Skyra (Siemens Medical Solutions, Erlangen, Germany) with a standard transmit–receive head coil in the General Hospital of People’s Liberation Army. Before the beginning of the scan, the subjects were instructed to prepare for the test (by removing magnetic objects, wearing shoes, and wearing earplugs, for example). The subjects were then instructed to lie supine on the MRI bed, and their heads were fixed with sponge and bandage. At the beginning of each scan, high-resolution T1-weighted structural images (176 slices) and three-dimensional gradient echo images were acquired using the following parameters: repetition time = 2 s, echo time = 30 ms, flip angle = 12°, field of view = 256 mm × 256 mm, matrix = 64 × 64, voxel size = 1 × 1 × 1 mm, and no slice gap. Resting fMRI data were acquired with one run of 8 min (240 images per session) using the following parameters: repetition time = 2 s, echo time = 30 ms, flip angle = 90°, field of view = 256 × 256 mm, matrix = 64 × 64, slice thickness = 3 mm, and slice gap = 1 mm. During the scan, the subjects were asked to close their eyes, keep their heads and body as steady and still as possible, and think about nothing. Data, such as heart rate and breathing, were collected at the same time. It is important for the subjects to remain awake during the scanning process, and so the operators communicated with the subjects through a microphone before each scan to remind them to stay awake. After each scan, the subjects were asked whether they had stayed awake during the scanning process.

### Data Processing

The resting-state fMRI images were preprocessed using SPM 12 software (University College London)^1^ and the CONN toolbox software version 18a (Neuroimaging Informatics Tools and Resources Clearinghouse)^2^, both of which are cross-platform software based on MATLAB (MathWorks, Inc., Natick, MA, United States). We selected the default preprocessing steps of CONN that include structural translation, segmentation, and normalization; functional realignment and unwarping; functional slice-timing correction; functional indirect segmentation and normalization; and outlier detection and smoothing. The first 10 volumes of the functional images were discarded to ensure the equilibration of the MRI data signal. The functional images were then registered to the middle volume of each subject to measure the degree of head movement, and the rotational and translational motion of subjects was limited to 2° or 2 mm in the *x*, *y*, and *z* axes, respectively. Frames showing more than 2° or 2 mm of head movement from one frame to the next were removed. In head movement processing, the mean frame-wise displacement (FD) was also calculated, and the subjects with the mean FD-Jenkinson >0.2 were excluded (Jenkinson et al., 2002). The structural images were normalized directly to the standard Montreal Neurological Institute (MNI)-152 space using EPI templates, with a voxel size of 3 × 3 × 3 mm. The functional images were normalized to the standard space indirectly using the corresponding structural images by which normalized bias correction was generated. A multiple regression was used to remove nuisance signals from the time series. A full nuisance regression including polynomial detrending in amplitude of low-frequency fluctuations was used to remove nuisance signals from the time series (Woletz et al., 2018). The cerebrospinal fluid signal, white matter signal, whole-brain signal, and six motion parameters were then eliminated. Subsequently, the images were spatially smoothed using a Gaussian filter with the full width at half maximum (FWHM) for 6 mm, and a band pass filter of 0.01–0.08 Hz was used to filter the data temporally (Behzadi et al., 2007; Whitfield-Gabrieli and Nieto-Castanon, 2012).

### Functional Connectivity Analysis

We used the CONN software to study the functional connectivity of the PC with other regions by region of interest to region of interest (ROI-to-ROI) analysis. All ROIs were drawn from Automated Anatomical Labeling (AAL) including 90 cortex ROIs and 26 cerebellar ROIs (Tzourio-Mazoyer et al., 2002). In the first-level analysis, the functional connectivity of different sources was assessed separately for each subject, and the mean time series of the seed point regions of the lateral PC was compared with that of the whole brain to generate a ROI-to-ROI diagram. The data processing method mainly included a general linear model convolved with typical hemodynamic response functions. In the second-level analysis, comparisons were made between subjects [SD > RW (1, −1)] based on a general linear model of random effects, and a seed level correction was performed for multiple comparisons (false discovery rate, *p* < 0.05) (Whitfield-Gabrieli and Nieto-Castanon, 2012).

### Behavioral Correlation Analysis

Visual Analog Scale has a good reliability and validity in the preliminary assessment of psychological states (Miller and Ferris, 1993; Huang et al., 2019), so we initially used VAS as first-level subjective measures of mood change before and after SD. We then used PVT to measure changes in psychomotor vigilance before and after SD. As shorter-duration PVT may be more sensitive to sleep loss, we chose the first 3 min of data as the final data for processing (Loh et al., 2004; Basner and Rubinstein, 2011). In order to further assess the functions of the PC, we used Spearman’s rank correlation to calculate the correlation coefficient between VAS changes and functional connectivity before and after SD. We thereafter studied the correlation coefficient between changes in PVT and functional connectivity. The false positive control of analysis was considered significant at *p* < 0.05.

## Results

### Initial Quality Assessment of the Data

During the experiments, no accidents or adverse events occurred, and no subjects were excluded for such reasons. In the preliminary processing of the functional magnetic resonance data, two subjects were excluded from all statistical analyses due to head movement exceeding the mean FD-Jenkinson >0.2 and a large number of frames with head movement greater than 2° or 2 mm. In the evaluation of the VAS, two people were excluded because the data were not saved due to errors in the questionnaire acquisition terminal. In the processing of PVT data, no subjects were excluded. A total of *n* = 28 subjects were included in the functional connectivity analysis. A total of *n* = 28 subjects were included in the behavioral analysis, and a total of *n* = 26 subjects were included in behavioral correlation analysis. The second-level correlation analysis between the change in functional connectivity and VAS included 26 subjects. The correlation analysis of the change in functional connectivity and PVT included 28 subjects.

### Behavioral Results

Demographic and Sleep Quality Index data were collected (Table 1). By paired sample *t*-test, the VAS showed significant changes before and after SD in levels of anxiety (*t* = 7.641, *p* < 0.001), attention (*t* = −2.87, *p* < 0.008), self-confidence (*t* = 6.986, *p* < 0.001), anger (*t* = 7.486, *p* < 0.001), and nervousness (*t* = −2.069, *p* = 0.049) of the participants (Table 1). PVT monitoring data were also statistically analyzed by paired sample *t*-test, identified significant differences in psychomotor vigilance before and after SD, especially in the mean response time (mean RT), and the fastest 10% fastest response time (Fastest 10% RT), the slowest 10% fastest response time (Slowest 10% RT).

**TABLE 1:** Demographic data, sleep quality index, and psychological traits (*n* = 28).

	RW	SD	*t*	*p*-value
Ages	24.482.57	–	–	–
Height	175.935.01	–	–	–
BMI	23.641.73	–	–	–
PSQI	3.371.19	–	–	–
Attention (VAS)	82.6914.85	56.5419.79	7.64	<0.001
Anxiety (VAS)	26.1515.51	35.7719.22	–2.87	0.008
Vigor (VAS)	83.4615.99	53.0819.34	6.99	<0.001
Self-confidence (VAS)	84.6214.76	59.6218.86	7.49	<0.001
Anger (VAS)	23.0813.79	30.3817.77	–2.07	0.049
Nervousness (VAS)	26.1518.78	33.0817.15	–2.09	0.047
Mean RT (PVT)	351.4235.65	373.9842.05	–4.84	0.001
Fastest 10% RT (PVT)	284.3827.18	312.5839.21	–4.40	<0.001
Slowest 10% RT (PVT)	431.5339.69	447.9441.64	–2.05	0.05
Lapse probability (PVT)	5.510.49	7.117.20	–0.97	0.34

*RW, rested wakefulness; SD, sleep deprivation; BMI, body mass index; PSQI, Pittsburgh Sleep Quality Index; VAS, Visual Analog Scale; RT, reaction time; PVT, Psychomotor Vigilance Test.*

**FIGURE 1:**
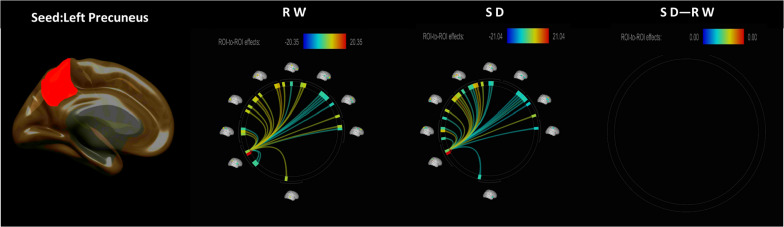
ROI-to-ROI functional connectivity of the left precuneus during the RW, SD, and SD > RW conditions. False discovery rate-corrected (*p* < 0.05) for ROI-to-ROI tests. ROI, region of interest; SD, sleep deprivation; RW, rested wakefulness.

**FIGURE 2:**
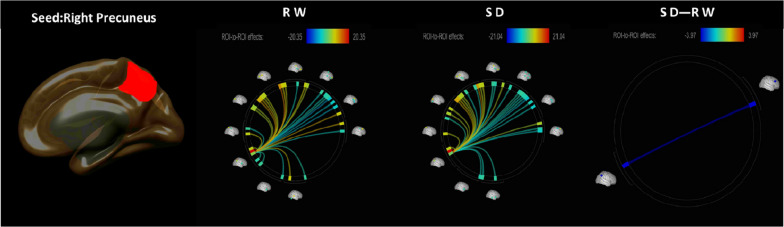
ROI-to-ROI functional connectivity of the right precuneus during the RW, SD, and SD > RW conditions. False discovery rate-corrected (*p* < 0.05) for ROI-to-ROI tests. ROI, region of interest; SD, sleep deprivation; RW, rested wakefulness.

The functional connectivity between the bilateral PC and the whole-brain ROIs in the RW and SD conditions is shown in Figures 1, 2. CONN software was used to calculate the changes in the functional connections between the PC and other seed regions of the brain before and after SD, and it revealed that the functional connections between the right PC and the right middle frontal gyrus (MFG) lobe were obviously weaken (Figure 2 and Table 2).

**TABLE 2:** ROI-to-ROI functional connectivity statistics for an individual seed region: comparisons between SD and RW scans (*t*-test).

Target region	AAL label	MNI center	*t*	Uncorrected *p*-value	FDR-corrected *p*-value
rPC	Right precuneus	9, −56, 44			
lPC	Left precuneus	−8, −56, 48			
Rmfg	Right middle frontal gyrus	37, 33, 34	−3.97	0.0005	0.0324

*ROI, region of interest; SD, sleep deprivation; RW, rested wakefulness; AAL, Automated Anatomical Labeling.*

**FIGURE 3:**
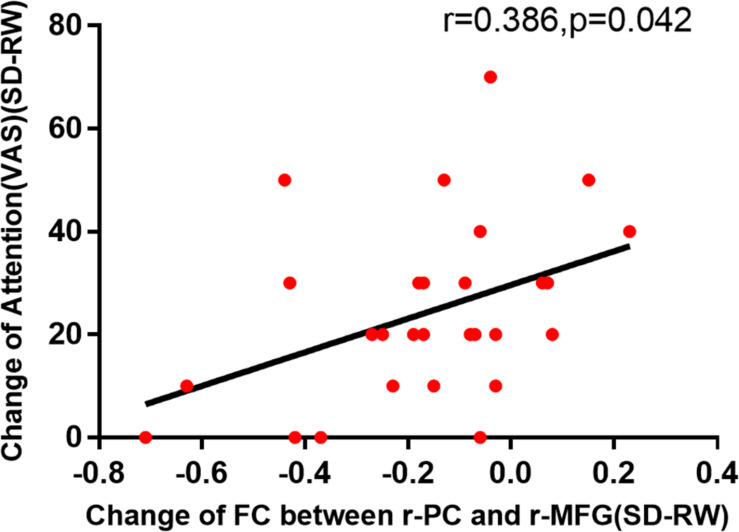
The change in functional connectivity was significantly correlated with the VAS of attention. VAS, Visual Analog Scale; SD, sleep deprivation; RW, rested wakefulness.

**FIGURE 4:**
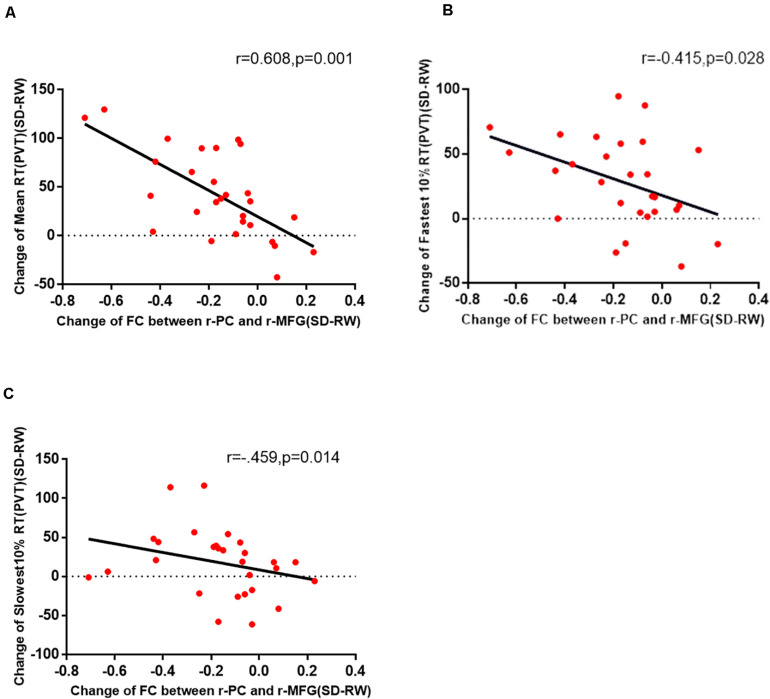
The change in functional connectivity between the right PC and the right MFG was significantly correlated with the mean RT **(A)**, fastest 10% RT **(B)**, and slowest 10% RT **(C)** of PVT. PC, precuneus; MFG, right middle frontal gyrus; PVT, psychomotor vigilance test; SD, sleep deprivation; RW, rested wakefulness; RT, response time.

In this experiment, there is a positive correlation between attention (VAS) and decrease of functional connectivity by Pearson correlation analysis to explore the possible correlations between VAS and the changes in functional connectivity of the right PC and the right MFG (Figure 3). Then, the Pearson correlation analysis between PVT and the changes in functional connectivity of the right PC and the right MFG also suggested that the decreased functional connectivity was significantly negatively correlated with an increased maximum 10% RT of PVT (Figure 4).

## Discussion

In this study, we assessed the effects of 36 h of SD on functional connections between the PC and other regions of the brain. The results showed that the functional connections between the right PC and the right MFG were significantly weakened after 36 h of acute SD. However, the changes of functional connectivity between the PC and other brain regions showed no significant statistical changes.

Previous studies have shown that the brain functions sensitive to SD are associated with substantial impairments in cognitive performance (especially attention and working memory), emotion, and regulation and memory abilities (Durmer and Dinges, 2005; Goel et al., 2009; Kelley et al., 2013; Louca and Short, 2014). On the one hand, the DMN is closely related to attention task execution under the SD condition. However, the DMN is not a homogeneous network, and SD has a dissociative effect on the functional connectivity of the DMN. The functional connectivity between the ventral DMN and the dorsal DMN is enhanced after SD. After SD, the decrease of functional connectivity of the dorsal DMN is associated with impairment of basic cognitive function and associated with RTs of PVT (Buckner and DiNicola, 2019). In the case of the PC, it is located in the dorsal region of the posterior medial parietal lobe. As a part of the dorsal DMN that is involved in a wide spectrum of attention processes (Vogt and Derbyshire, 2009), the PC acts as an attention information integration center that organizes information from different regions of the brain (Lin et al., 2011). In contrast, some studies suggest that the inferior frontal junction comprising posterior aspects of the inferior frontal sulcus may be an important node that interacts between the ventral attention network (VAN) and the Dorsal Attention Network (DAN) (Asplund et al., 2010). Some studies propose that the right MFG may be the node that links the dorsal and ventral networks (Fox et al., 2006). Interruption of the ongoing process of the dorsal network while focusing on a new task-related external stimulus is not recommended (Corbetta et al., 2008). Furthermore, a positive function of the MFG consisted of attention tasks, suggesting that it is a key feature in sustained attention (Neale et al., 2015), and that the MFG is an important part of the attention network (Gogulski et al., 2017). Given the consistency of multiple functions between the two brain regions and considering that the functional connection between the right PC and the right MFG is the only significant change between the PC and the rest of the brain before and after SD, it is reasonable to presume that the functional connections between the PC and the MFG were probable to be attention-related.

Thereafter, the correlation analysis between the changes of the brain functional VAS and the changes between the right PC and the right MFG preliminary supported this hypothesis and suggested that the changes in attention were significantly correlated with the changes in functional connectivity between the two brain regions (Figure 2). VAS is a simple but effective subjective assessment method (Huang et al., 2019). Although the sample size was small and there may be bias, the results it presents provide a relatively clear direction for further research on brain function. Changes in functional connectivity led to no obvious related changes in anxiety, vigor, self-confidence, anger, and nervousness. The PC and MFG are important nodes of their respective brain networks, which are related to a variety of brain functions. The PC is associated with a variety of brain functions, not least because the PC region of the brain is an integrated region of neural networks, especially the DMN, where the brain integrates and distributes signals. The MFG is also associated with a variety of functions, including attention, speech, mood, and wakefulness (Kelley et al., 2013). However, the range of brain functional tasks that may be undertaken by the connection between the right PC and the right MFG will be significantly reduced. This will be attributed to inconsistent bilateral brain function. The left and right MFG have clear functional differences, and the functional asymmetry in MFG is concerned with different brain networks (Song et al., 2019); the left MFG has been found to be associated with working memory, memory retrieval, social perception, and emotional regulation (Zhang et al., 2003; Ochsner and Gross, 2005; Wang et al., 2018). The right MFG plays an important role in sustained attention, and the hemispheric specialization of attention function is caused by the incongruous interhemispheric interaction between the left and right MFG. Therefore, the results from correlation analysis of the changes between various VAS and the functional connectivity preliminarily verified our hypotheses.

To further confirm our inference on the basis of the preliminary results, we used a classic tool for monitoring and evaluating SD and further analyzed the correlation between the PVT and changes in the functional connection between the right PC and the right MFG. We found that there was a moderate correlation between the fastest 10% RT (*r* = −0.415, *p* = 0.028) and the functional connection of two regions and a strong correlation between the mean 10% RT and the functional connection (*r* = −0.608, *p* = 0.001). As a classic detection method of psychomotor vigilance, the PVT measures changes in RT to visual stimuli to measure attention and vigilance (Warm et al., 2008). It is a very sensitive measure of vigilant attention as well as the degree of acute and chronic sleep disorder and circadian misalignment (Goel et al., 2009; Basner et al., 2015). With negligible aptitude and learning effects, the PVT is probably the most widely used measure of alertness (Lim and Dinges, 2008; Basner et al., 2015).

In this study, functional connectivity between the right PC and the right MFG after acute SD was associated with a change in attention VAS scores and a change in RTs in the PVT. This suggested that the right PC and the right MFG were involved in the function of attention in their respective networks, that there probably exists a connection point between the two networks, and that there was an attention-related brain network connection between the right PC and the right MFG.

## Limitations of the Study

Our experiments focused on the effects of acute SD on young people in modern life, given that the majority of work with sleep disorders in China is done by young men. Therefore, the experimental subjects are young men. On the other hand, sleep problems are also prominent in women. We will continue to improve relevant studies in the following studies.

## Conclusion

In conclusion, taken together, our resting-state fMRI and behavior results suggest that the functional connections between the right PC and the right MFG before and after SD were decreased, and that the decreased functional connections were significantly correlated with decreased attention. We conclude that the right PC and the right MFG play an important role in the maintenance of attention, and that there may well be a functional connective basis for the maintenance of attention between them, which is an important node for the maintenance of brain attention function.

## Data Availability Statement

The datasets generated for this study are available on request to the corresponding author.

## Ethics Statement

The studies involving human participants were reviewed and approved by the Beihang University. The patients/participants provided their written informed consent to participate in this study.

## Author Contributions

BL contributed to performing the experiments and acquisition, analysis, interpretation of the data, and drafted the article. LZ, YZ, and YC contributed to performing the experiments and acquisition of the data. JP reviewed the literature. YS and XZ were the guarantors of this study. All authors have made substantial contribution to this work and approved it for publication.

## Conflict of Interest

The authors declare that the research was conducted in the absence of any commercial or financial relationships that could be construed as a potential conflict of interest.

## Footnotes

1 http://www.fil.ion.ucl.ac.uk/spm/

2 http://www.nitrc.org/projects/conn
